# Supplementary material for: A buprenorphine depot formulation provides effective sustained post-surgical analgesia for 72 h in mouse femoral fracture models
Source: Sci Rep. 2023 Mar 7;13:3824. doi: 10.1038/s41598-023-30641-9 (PMC9992384; doi:10.1038/s41598-023-30641-9)
Supplement: Supplementary file 1 — Supplementary Information. [file 41598_2023_30641_MOESM1_ESM.pdf]

Supplementary Information

A buprenorphine depot formulation provides effective sustained post-surgical analgesia for 72h in mouse femoral fracture models

Angelique Wolter[1,2,3#], Christian H. Bucher[4,5], Sebastian Kurmies[1,2], Viktoria Schreiner[6], Frank Konietschke[5,7], Katharina Hohlbaum[3,8], Robert Klopfleisch[9], Max Löhning[1,2], Christa Thöne-Reineke[3], Frank Buttgereit[1,2], Jörg Huwyler[6], Paulin Jirkof[10], Anna E. Rapp[1,2,11*], Annemarie Lang[1,2,12*#]

Affiliations:

[1]Department of Rheumatology and Clinical Immunology, Charité – Universitätsmedizin Berlin, Corporate member of Freie Universität Berlin and Humboldt-Universität zu Berlin, Berlin, Germany

[2]German Rheumatism Research Centre (DRFZ), a Leibniz Institute, Berlin, Germany

[3]Institute of Animal Welfare, Animal Behavior and Laboratory Animal Science, Department of Veterinary Medicine, Freie Universität Berlin, Berlin, Germany

[4]Julius Wolff Institute, Charité – Universitätsmedizin Berlin, Berlin, Germany

[5]Berlin Institute of Health Center for Regenerative Therapies (BCRT), Charité – Universitätsmedizin Berlin, Berlin, Germany

[6]Division of Pharmaceutical Technology, Department of Pharmaceutical Sciences, University of Basel, Basel, Switzerland

[7]Institute of Biometry and Clinical Epidemiology, Charité-Universitätsmedizin Berlin, Corporate Member of Freie Universität Berlin and Humboldt-Universität zu Berlin, Berlin, Germany

[8]German Centre for the Protection of Laboratory Animals (Bf3R), German Federal Institute for Risk Assessment (BfR), Berlin, Germany

[9]Institute of Veterinary Pathology, Department of Veterinary Medicine, Freie Universität Berlin, Berlin, Germany

[10]Office for Animal Welfare and 3Rs, University of Zurich, Zurich, Switzerland

[11]Dr. Rolf M. Schwiete Research Unit for Osteoarthritis, Department of Orthopedics (Friedrichsheim), University Hospital Frankfurt, Goethe University, Frankfurt, Germany

[12]Departments of Orthopaedic Surgery and Bioengineering, University of Pennsylvania, PA, United States

[#]Correspondences:

Angelique Wolter, Institute of Animal Welfare, Animal Behavior and Laboratory Animal Science, Department of Veterinary Medicine, Freie Universität Berlin, Berlin, Germany

a.wolter@fu-berlin.de

Annemarie Lang, Departments of Orthopaedic Surgery and Bioengineering, University of Pennsylvania, PA, United States

Annemarie.lang@pennmedicine.upenn.edu

Health monitoring was carried out in accordance with FELASA recommendations.

Species: mouse
Test animals: bedding sentinels
Housing type: semi-barrier, limited access, protective clothing, IVC
Abbreviations: CULT = Culture; PCR = Polymerase Chain Reaction; IFA = Immunofluorescence Assay; PATH = Gross Pathology; MICR = Microscopy; POS = Positive; NEG = Negative; NT = Not Tested
Laboratory: GIM Gesellschaft für innovative Mikroökologie mbH, Waldheimstraße 47, 14552 Berlin

The following table summarizes the testing results. The study was caried out in between the six months between “latest results” and “previous results”.

	Test frequency	Latest results	Previous results	Testing methods
Number of animals tested		n = 4	n = 4	
**Viruses**				
Mouse rotavirus (EDIM)	6 months	NEG	NEG	IFA
Mouse hepatitis virus (MHV)	6 months	NEG	NEG	IFA
Murine norovirus (MNV)	6 months	POS(4)	POS(4)	IFA
Mouse parvovirus (MPV)	6 months	NEG	NEG	IFA
Minute virus of mice (MVM)	6 months	NEG	NEG	IFA
Theiler´s murine encephalomyelitis virus (TMEV)	6 months	NEG	NEG	IFA
Lymphocytic choriomeningitis virus (LCMV)	annually	NEG	NT	IFA
Mouse adenovirus 1/2 (Fl/K87)	annually	NEG	NT	IFA
Mousepox (ectromelia) virus (EV)	annually	NEG	NT	IFA
Pneumonia virus of mice (PVM)	annually	NEG	NT	IFA
Reovirus type 3 (Reo)	annually	NEG	NT	IFA
Sendai virus (SV)	annually	NEG	NT	IFA
**Bacteria**				
Rodentibacter spp.	6 months	NEG	NEG	CULT
Streptococci spp. ß-haemolytic/not group D	6 months	NEG	NEG	CULT
Streptococcus pneumoniae	6 months	NEG	NEG	CULT
Helicobacter spp.	6 months	POS(2)	POS(2)	PCR
Corynebacterium kutscheri	annually	NT	NT	CULT
Citrobacter rodentium	annually	NT	NT	CULT
Salmonella spp.	annually	NT	NT	CULT
Streptobacillus moniliformis	annually	NT	NT	PCR
Clostridium piliforme	annually	NT	NT	IFA
Mycoplasma pulmonis	annually	NT	NT	IFA
**Parasites**				
Ectoparasites	6 months	NEG	NEG	MICR
Endoparasites	6 months	POS(6)	POS(4)	MICR
Aspiculuris spp.	6 months	NEG	NEG	MICR
Syphacia spp.	monthly	NEG	NEG	MICR
Chilomastix spp.	6 months	NEG	NEG	MICR
Coccidia spp.	6 months	NEG	NEG	MICR
Entamoeba spp.	6 months	NEG	NEG	MICR
Giardia spp.	6 months	NEG	NEG	MICR
Spironucleus muris	6 months	NEG	NEG	MICR
Trichomonas spp.	6 months	POS(2)	NEG	MICR
other flagellates	6 months	POS(4)	POS(4)	MICR
**Pathological lesions**	6 months	NEG	NEG	PATH

Exclusion of mice and data

Three male mice were excluded before the beginning of the study due to one sudden death and two urethral obstructions resulting in n = 9 in the following groups: Tramadol rigid, Tramadol flexible and BUP-Depot (Fig. 1A). The following data had to be additionally excluded from analyses: water intake of one cage at 24h after osteotomy due to a leaking bottle (male mice, BUP-Depot, flexible); one male mouse (BUP-Depot, rigid) from composite score and CatWalk analyses, as this animal received an additional injection of Temgesic at 24h post-osteotomy; one female mouse (BUP-Depot, flexible) from stride length analysis (CatWalk) as an technical artifact at 48h post-osteotomy; one female mouse (BUP-Depot, flexible) from limping score analyses at all time points due short-term nerve damage (transient dragging); four samples from *ex-vivo* µCT, histology and immunofluorescence (Tramadol rigid (male), Tramadol flexible (male), Tramadol rigid (female), BUP-Depot flexible (female)) due to insufficient fixation which was not visible until sample collection. In some samples, immunofluorescence staining was not sufficient for analysis and resulted in the exclusion of these samples.

Supplementary Results

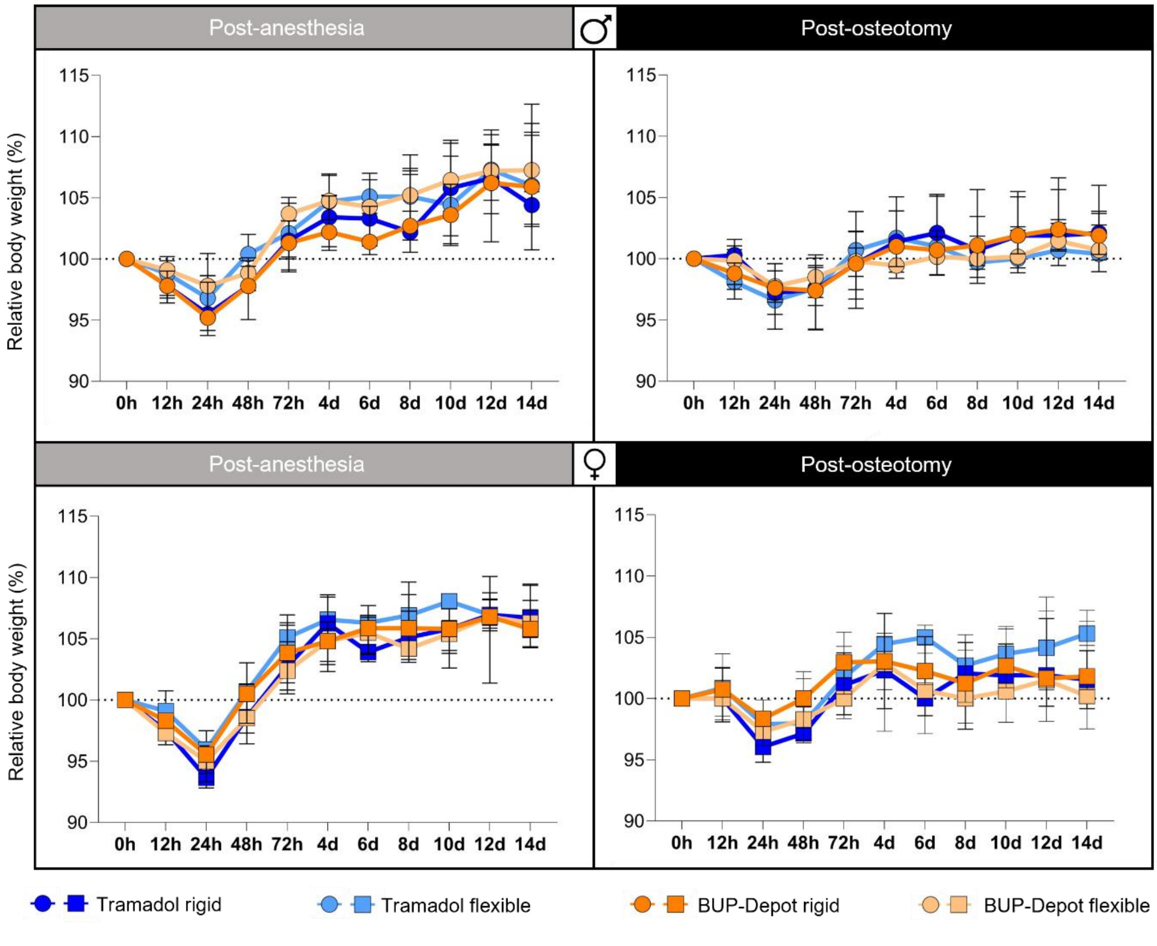

Figure S1: Relative body weight development over 14 days post-anesthesia and post-osteotomy. Body weight was measured at 12h, 24h, 48h and 72h post-anesthesia and post-osteotomy and afterwards every other day until osteotomy/euthanasia. Body weight was normalized to the initial value (0h = 100%). All graphs show median with interquartile range for n= 9-10. No statistical analysis was performed.

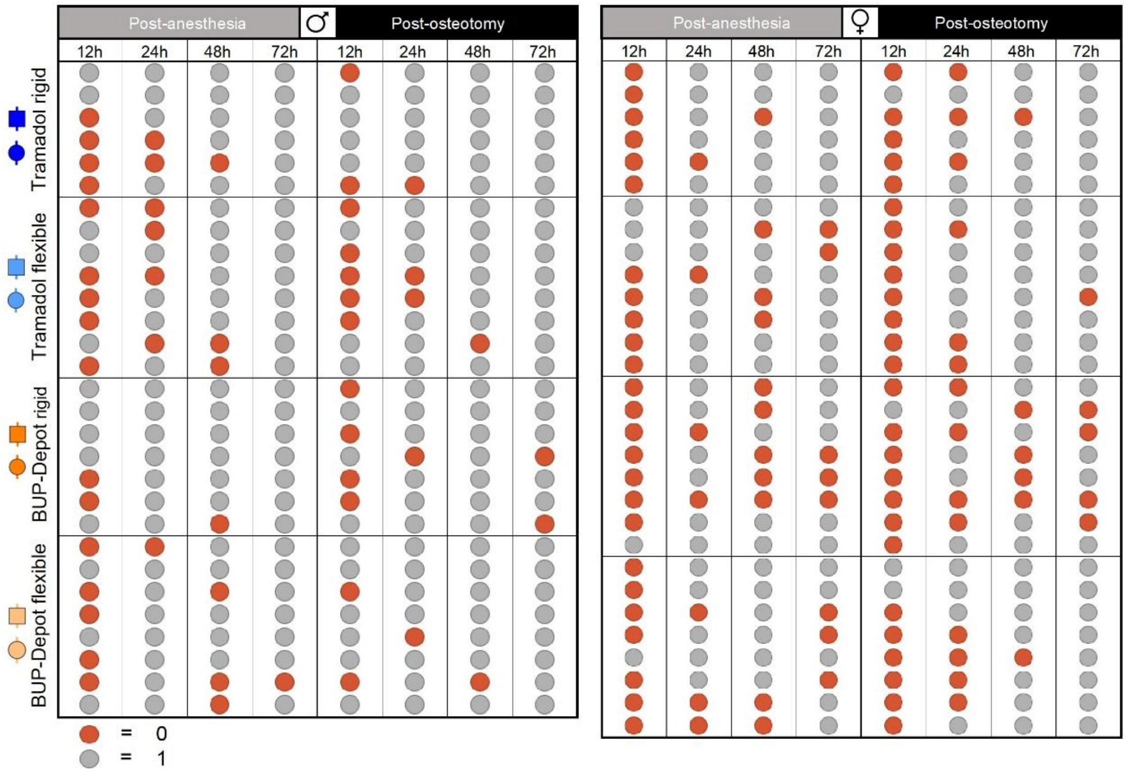

Figure S2: Defecation during the assessments post-anesthesia and post-osteotomy. Mice were closely monitored for defecation during the assessments at 12h, 24h, 48h and 72h post-anesthesia and post-osteotomy (0 = no defecation, 1 = defecation). n = 6-8.

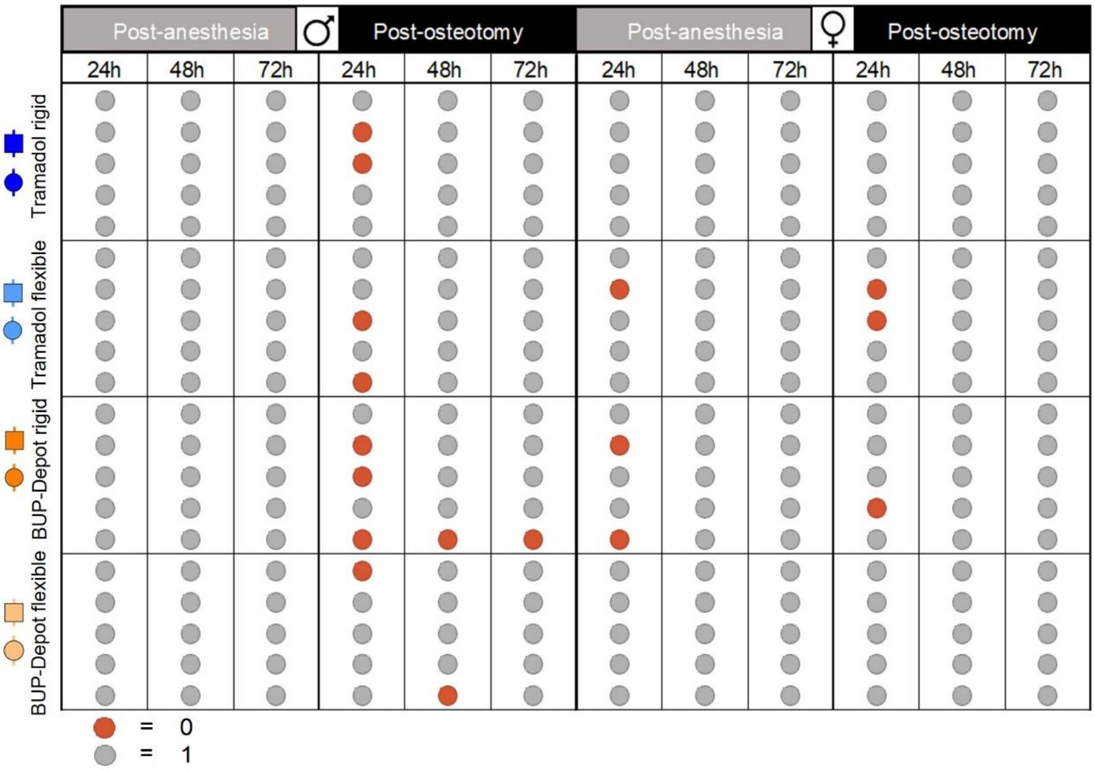

Figure S3: Explorative behavior post-anesthesia and post-osteotomy. Explorative behavior was monitored per cage at 24h, 48h and 72h post-anesthesia and post-osteotomy (0 = no explorative behavior, 1 = explorative behavior). n = 5 based on cages (pair housing).

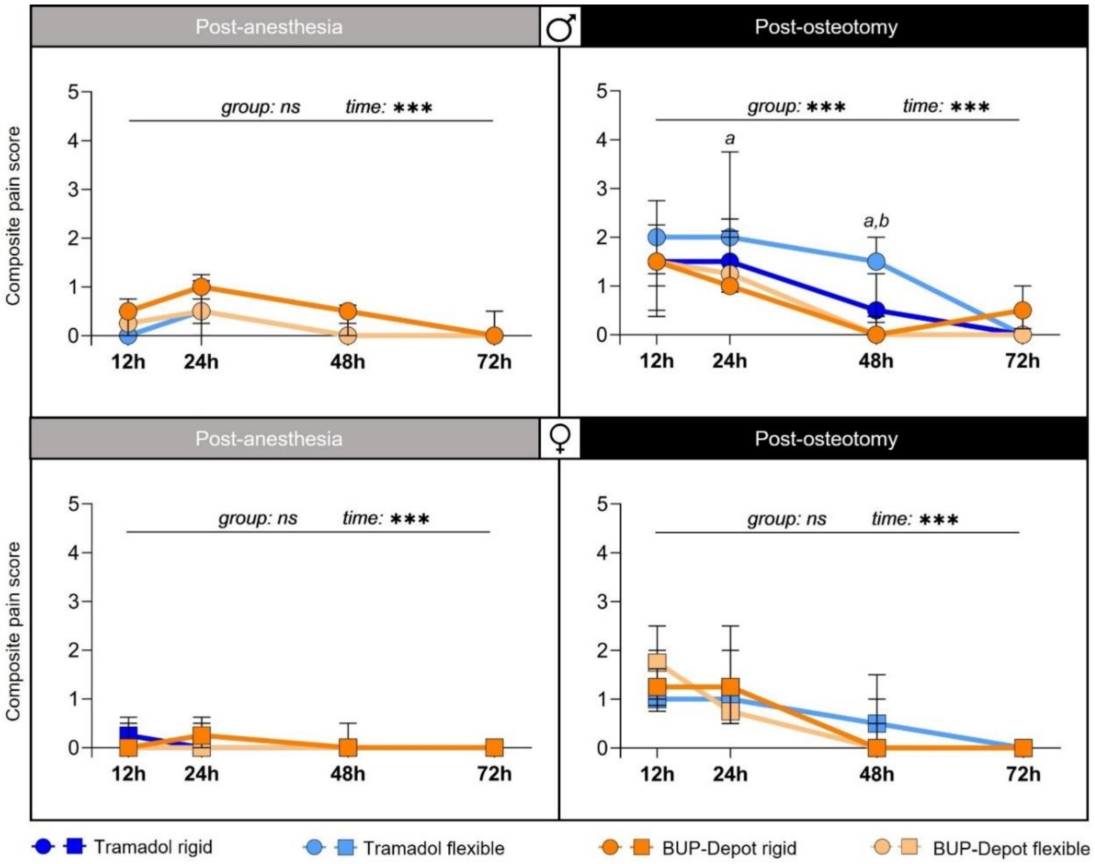

Figure S4: Composite pain scores post-anesthesia and post-osteotomy. The composite pain score was measured at 12h, 24h, 48h and 72h post-anesthesia and post-osteotomy. All graphs show median with interquartile range for n= 9-10. Non-parametric ANOVA-type test - main effects of time and of group are represented in the graphs; exact *p*-values are listed in Supplementary Table S4-S4.3; **p < 0.05, ***p < 0.001*. To determine group differences Dunn's post hoc test with Bonferroni correction was performed. *a* - significant difference Tramadol flexible vs. BUP-Depot flexible; *b* - significant difference Tramadol flexible vs. BUP-Depot rigid.

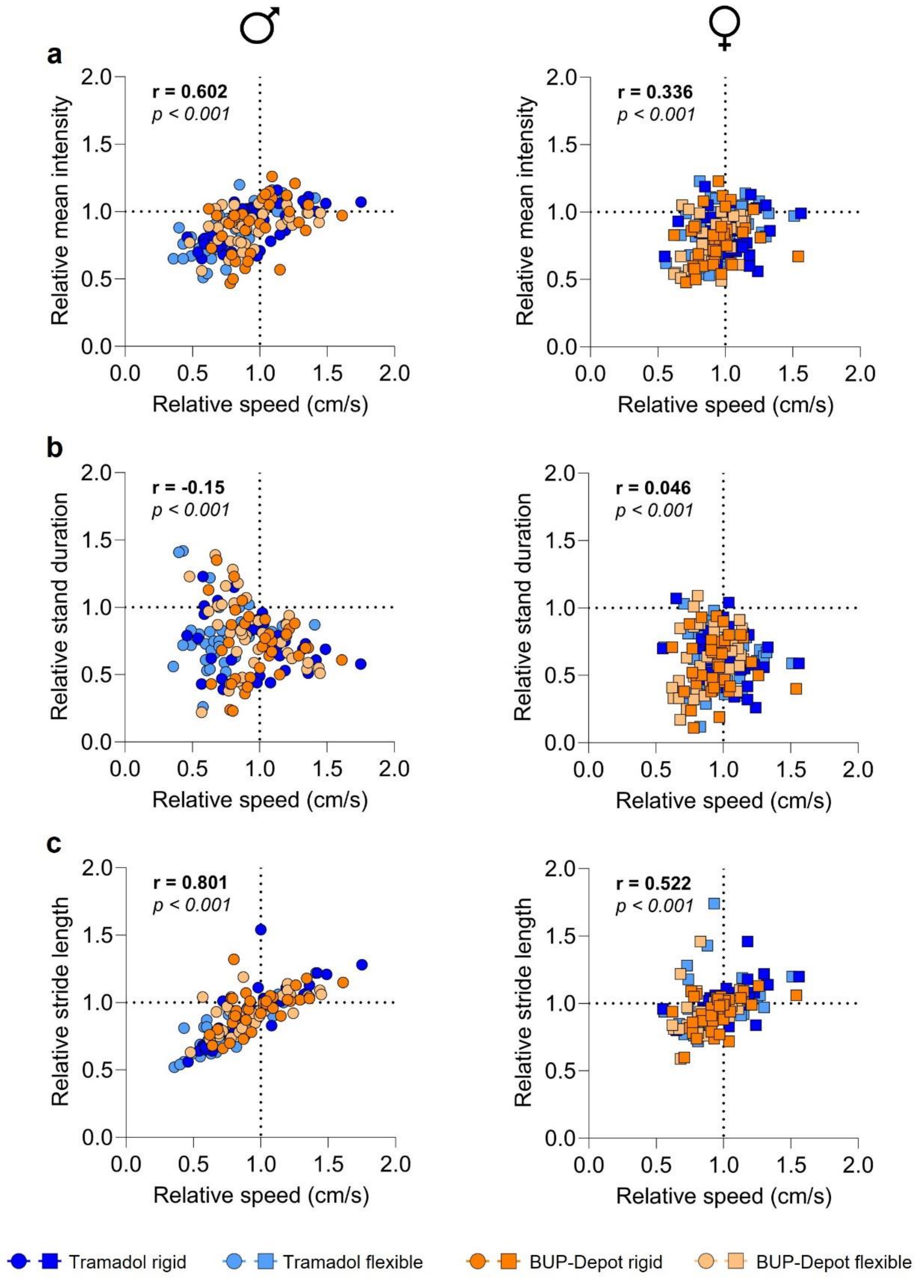

Figure S5: Spearman correlation between relative velocity and (a) relative mean intensity, (b) relative stand duration and (c) relative stride length. All parameters were measured at 24h, 48h, 72h and 10d post-osteotomy using the Noldus CatWalk XT. Spearman correlation was carried out combining data from all four time points post-osteotomy. Correlation was carried out in Prism (V9).

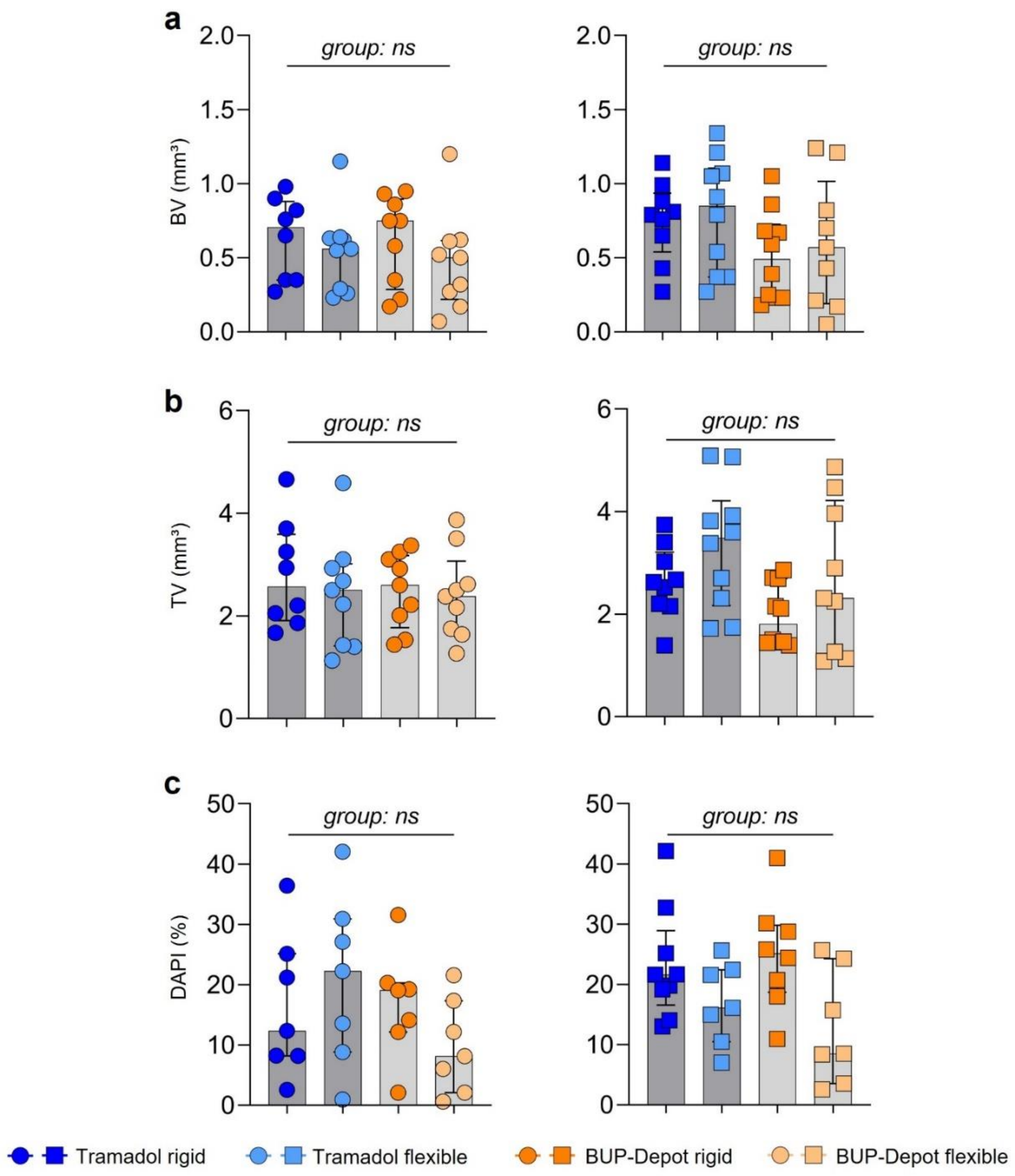

Figure S6. (a) Bone volume (BV) (mm³), (b) total volume (TV) (mm³) and (c) DAPI (%) were measured 14d post-osteotomy. All graphs show median with interquartile range for n = 8-10 (**a-b**) and n = 6-9 (**c**). To determine group differences, Kruskal-Wallis, and Dunn's post hoc test with Bonferroni correction was performed in RStudio; exact *p*-values are listed in Supplementary Table **S11-S13.1**.

Supplemental Statistics

Table S1: *p*-values for body weight, *nonparametric analysis of longitudinal data (nparLD) (F1-LD-F1 design)*

	♂ post-anesthesia	♂ post-osteotomy	♀ post-anesthesia	♀ post-osteotomy
Group	0.631	0.887	**0.0134**	0.367
Time	**< 0.001**	**< 0.001**	**< 0.001**	**< 0.001**
Group:Time	0.699	0.841	0.865	0.740

Table S1.1: Body weight, females (♀), post-anesthesia, *Kruskal-Wallis test*

	p-value	chi-squared	df
12h post-anesthesia	**0.025**	9.329	3
24h post-anesthesia	0.051	7.784	3
48h post-anesthesia	0.074	6.925	3
72h post-anesthesia	0.351	3.279	3

Table S1.2: *p*-values for body weight, females (♀), post-anesthesia, *Dunn's post hoc test (Bonferroni correction), 12h post-anesthesia*

	♀, Tramadol flexible	♀, BUP-Depot rigid	♀, BUP-Depot flexible
♀, Tramadol rigid	0.060	0.311	1.000
♀, BUP-Depot rigid	1.000	-	
♀, BUP-Depot flexible	**0.031**	0.186	

Table S1.3: *p*-values for body weight, females (♀), post-anesthesia, *Dunn's post hoc test (Bonferroni correction), 24h post-anesthesia*

	♀, Tramadol flexible	♀, BUP-Depot rigid	♀, BUP-Depot flexible
♀, Tramadol rigid	**0.017**	0.305	0.414
♀, BUP-Depot rigid	0.765	-	
♀, BUP-Depot flexible	0.589	1.000	

Table 2: *p*-values for food intake, *nonparametric analysis of longitudinal data (nparLD) (F1-LD-F1 design)*

	♂ post-anesthesia	♂ post-osteotomy	♀ post-anesthesia	♀ post-osteotomy
Group	0.655	0.824	0.655	0.582
Time	**< 0.001**	**< 0.001**	**< 0.001**	**< 0.001**
Group:Time	0.383	0.714	**0.027**	0.638

Table S3: *p*-values for water intake, *nonparametric analysis of longitudinal data (nparLD) (F1-LD-F1 design)*

	♂ post-anesthesia	♂ post-osteotomy	♀ post-anesthesia	♀ post-osteotomy
Group	0.410	0.515	0.512	0.272
Time	**< 0.001**	**< 0.001**	**< 0.001**	**< 0.001**
Group:Time	0.944	0.861	0.487	**0.039**

Table S4: *p*-values for the composite pain score, *nonparametric analysis of longitudinal data (nparLD) (F1-LD-F1 design)*

	♂ post-anesthesia	♂ post-osteotomy	♀ post-anesthesia	♀ post-osteotomy
Group	0.184	**< 0.001**	0.678	0.334
Time	**< 0.001**	**< 0.001**	**< 0.001**	**< 0.001**
Group:Time	0.970	**0.006**	0.276	0.663

Table S4.1: Composite pain score males (♂), post-osteotomy, *Kruskal-Wallis test*

	p-value	chi-squared	df
12h post-osteotomy	0.182	4.861	3
24h post-osteotomy	**0.025**	9.316	3
48h post-osteotomy	**0.001**	15.878	3
72h post-osteotomy	0.234	4.271	3

Table S4.2: *p*-values for the composite pain score, post-osteotomy, *Dunn's post hoc test (Bonferroni correction), 24h post-osteotomy*

	♂, Tramadol flexible	♂, BUP-Depot rigid	♂, BUP-Depot flexible
♂, Tramadol rigid	0.062	1.000	1.000
♂, BUP-Depot rigid	0.051	-	
♂, BUP-Depot flexible	**0.020**	1.000	

Table S4.3: *p*-values for the composite pain score, post-osteotomy, *Dunn's post hoc test (Bonferroni correction), 48h post-osteotomy*

	♂, Tramadol flexible	♂, BUP-Depot rigid	♂, BUP-Depot flexible
♂, Tramadol rigid	0.075	0.322	1.000
♂, BUP-Depot rigid	**0.001**	-	
♂, BUP-Depot flexible	**0.009**	0.941	

Table S5: *p*-values for the delta composite pain score, *nonparametric analysis of longitudinal data (nparLD) (F1-LD-F1 design)*

	♂ post-osteotomy	♀ post-osteotomy
Group	**< 0.001**	0.687
Time	**< 0.001**	**< 0.001**
Group:Time	0.081	0.383

Table S5.1: Delta composite pain score, males (♂), post-osteotomy, *Kruskal-Wallis test*

	p-value	chi-squared	df
12h post-osteotomy	0.056	7.561	3
24h post-osteotomy	**0.031**	8.909	3
48h post-osteotomy	**< 0.001**	19.397	3
72h post-osteotomy	0.278	3.847	3

Table S5.2: *p*-values for the delta composite pain score, *Dunn's post hoc test (Bonferroni correction), 24h post-osteotomy*

	♂, Tramadol flexible	♂, BUP-Depot rigid	♂, BUP-Depot flexible
♂, Tramadol rigid	**0.032**	1.000	1.000
♂, BUP-Depot rigid	**0.042**	-	
♂, BUP-Depot flexible	0.077	1.000	

Table S5.3: *p*-values for the delta composite pain score, *Dunn's post hoc test (Bonferroni correction), 48h post-osteotomy*

	♂, Tramadol flexible	♂, BUP-Depot rigid	♂, BUP-Depot flexible
♂, Tramadol rigid	**0.018**	0.377	1.000
♂, BUP-Depot rigid	**< 0.001**	-	
♂, BUP-Depot flexible	**0.004**	0.710	

Table S6: *p*-values for the limping score, *nonparametric analysis of longitudinal data (nparLD) (F1-LD-F1 design)*

	♂ post-osteotomy	♀ post-osteotomy
Group	0.436	0.108
Time	**< 0.001**	**0.018**
Group:Time	0.600	0.612

Table S7: *p*-values for CatWalk - relative velocity, *nonparametric analysis of longitudinal data (nparLD) (F1-LD-F1 design)*

	♂ post-anesthesia	♂ post-osteotomy	♀ post-anesthesia	♀ post-osteotomy
Group	0.266	**0.034**	0.563	**0.035**
Time	**0.003**	**< 0.001**	**0.007**	**< 0.001**
Group:Time	0.662	0.133	0.530	0.092

Table S7.1: CatWalk - relative velocity, males (♂), post-osteotomy, *Kruskal-Wallis test*

	p-value	chi-squared	df
24h post-osteotomy	**0.052**	7.735	3
48h post-osteotomy	**0.038**	8.428	3
72h post-osteotomy	0.097	6.316	3
10d post-osteotomy	0.110	6.035	3

Table S7.2: *p*-values for CatWalk - relative velocity, males (♂), post-osteotomy, *Dunn's post hoc test (Bonferroni correction), 24h post-osteotomy*

	♂, Tramadol flexible	♂, BUP-Depot rigid	♂, BUP-Depot flexible
♂, Tramadol rigid	0.119	1.000	1.000
♂, BUP-Depot rigid	**0.034**	-	
♂, BUP-Depot flexible	0.101	1.000	

Table S7.3: *p*-values for CatWalk - relative velocity, males (♂), post-osteotomy, *Dunn's post hoc test (Bonferroni correction), 48h post-osteotomy*

	♂, Tramadol flexible	♂, BUP-Depot rigid	♂, BUP-Depot flexible
♂, Tramadol rigid	0.053	1.000	1.000
♂, BUP-Depot rigid	**0.029**	-	
♂, BUP-Depot flexible	0.157	1.000	

Table S7.4: CatWalk - relative velocity, females (♀), post-osteotomy, *Kruskal-Wallis test*

	p-value	chi-squared	df
24h post-osteotomy	**0.009**	11.470	3
48h post-osteotomy	0.259	4.026	3
72h post-osteotomy	0.180	4.894	3
10d post-osteotomy	0.166	5.088	3

Table S7.5: *p*-values for CatWalk - relative velocity, females (♀), post-osteotomy, *Dunn's post hoc test (Bonferroni correction), 24h post-osteotomy*

	♀, Tramadol flexible	♀, BUP-Depot rigid	♀, BUP-Depot flexible
♀, Tramadol rigid	0.074	0.162	**0.003**
♀, BUP-Depot rigid	1.000	-	
♀, BUP-Depot flexible	0.855	0.583	

Table S8: *p*-values for CatWalk - relative mean intensity, *nonparametric analysis of longitudinal data (nparLD) (F1-LD-F1 design)*

	♂ post-osteotomy	♀ post-osteotomy
Group	0.489	0.653
Time	**< 0.001**	**< 0.001**
Group:Time	0.736	0.096

Table S9: *p*-values for CatWalk - relative stand duration, *nonparametric analysis of longitudinal data (nparLD) (F1-LD-F1 design)*

	♂ post-osteotomy	♀ post-osteotomy
Group	0.821	0.926
Time	**< 0.001**	**0.005**
Group:Time	0.692	0.468

Table S10: *p*-values for CatWalk - relative stride length, *nonparametric analysis of longitudinal data (nparLD) (F1-LD-F1 design)*

	♂ post-osteotomy	♀ post-osteotomy
Group	**0.024**	0.328
Time	**< 0.001**	0.053
Group:Time	0.175	0.580

Table S10.1: CatWalk - relative stride length, males (♂), post-osteotomy, *Kruskal-Wallis test*

	p-value	chi-squared	df
24h post-osteotomy	**0.044**	8.084	3
48h post-osteotomy	0.119	5.845	3
72h post-osteotomy	0.112	5.987	3
10d post-osteotomy	0.055	7.595	3

Table S10.2: *p*-values for CatWalk - relative stride length, males (♂), *Dunn's post hoc test (Bonferroni correction), 24h post-osteotomy*

	♂, Tramadol flexible	♂, BUP-Depot rigid	♂, BUP-Depot flexible
♂, Tramadol rigid	0.606	0.423	1.000
♂, BUP-Depot rigid	**0.018**	-	
♂, BUP-Depot flexible	0.151	1.000	

Table S11: *Ex-vivo* µCT: BV/TV, BV, TV, *Kruskal-Wallis test*

	♂			♀		
	p-value	chi-squared	df	*p*-value	chi-squared	df
BV/TV (%)	0.947	0.369	3	0.263	3.983	3
BV (mm^3)	0.400	2.947	3	0.248	4.124	3
TV (mm^3)	0.848	0.806	3	0.081	6.728	3

Table S12: Histology (Movat's pentachrome), *Kruskal-Wallis test*

	♂			♀		
	p-value	chi-squared	df	*p*-value	chi-squared	df
Relative bone fraction (%)	0.907	0.556	3	0.089	6.521	3
Relative cartilage fraction (%)	0.375	3.109	3	0.165	5.094	3

Table S13: Immunofluorescence, *Kruskal-Wallis test*

	♂			♀		
	p-value	chi-squared	df	*p*-value	chi-squared	df
Endomucin (%)	0.241	6.728	3	**0.045**	8.066	3
DAPI (%)	0.281	3.823	3	0.069	7.079	3

Table S13.1: Endomucin (%), females (♀), *Dunn's post hoc test (Bonferroni correction)*

	♀, Tramadol flexible	♀, BUP-Depot rigid	♀, BUP-Depot flexible
♀, Tramadol rigid	0.100	1.000	0.223
♀, BUP-Depot rigid	0.083	-	
♀, BUP-Depot flexible	1.000	0.186	
